# The abnormality of thyroid hormones in patients with type A hepatic encephalopathy

**DOI:** 10.18632/oncotarget.18869

**Published:** 2017-06-29

**Authors:** Lin Wang, Wanyou Yu, Wukui Cao, Wei Lu

**Affiliations:** ^1^ Liver Research Center, Beijing Friendship Hospital, Capital Medical University, National Clinical Research Center of Digestive Diseases, Beijing, China; ^2^ Department of ICU, Tianjin City Second People’s Hospital, Tianjin, China

**Keywords:** hepatic encephalopathy, acute liver failure, thyroid hormones, low TSH, inpatient survival

## Abstract

Abnormality of thyroid hormones in liver diseases is common, but data is lacking in patients with type A hepatic encephalopathy (HE). The present study was aimed to determine whether there was an abnormality in thyroid hormones among patients with type A HE. We measured the levels of thyroid hormones in 36 acute liver failure (ALF) patients with type A HE and in 29 acute liver injury patients (international normalized ratio, INR ≥ 1.5) without encephalopathy as control. The clinical parameters associated with abnormality of thyroid hormones were evaluated. ALF patients with type A HE exhibited decreased TSH levels compared to patients without encephalopathy (0.17 *vs* 1.08 μIU/mL, *P* < 0.001). There was no difference in T3 and T4 levels (both total and free) between the two groups. The logistic regression analysis identified type A HE as an independent related factor for the occurrence of low TSH (Odds Ratio = 12.32) in patients with ALF. Correlation analysis showed that there was an inverse correlation between TSH level and the grade of encephalopathy (r = −0.795). Furthermore, patients with low TSH depicted poor survival rate than those with normal TSH level (29.3% *vs* 44.1%, *P* = 0.003). Patients with type A HE exhibited subclinical central hypothyroidism, and had significant decreased TSH level, which had inverse correlation with the grade of encephalopathy. The reduced TSH was associated with poor survival rate.

## INTRODUCTION

Liver plays an important role in the metabolism of thyroid hormones. Therefore, patients with acute or chronic liver injury is often depicted abnormality of thyroid hormones, which is associated with liver dysfunction and poor prognosis [1–4]. However, data about alteration of thyroid hormones in patients with hepatic encephalopathy (HE) are scanty.

HE is a common complication of severe liver disease, which is grouped into three categories: type A is associated with acute liver failure (ALF), type B is associated with portal-systemic bypass without liver disease, and type C is HE that occurs in cirrhotic patients. Studies on thyroid hormones in patients with type C HE had been reported. T3 and T4 levels were lower in cirrhotic patients with type C HE compared with cirrhosis patients without HE [5, 6]. However, there is no report on alteration of thyroid hormones in patients with type A HE. The pathogenesis and manifestation of type A and type C HE are different [7, 8]. It is not clear whether type A HE has any effect on thyroid function. Type A HE is a life-threatening critical illness with high mortality. To explore alteration of thyroid hormones in type A HE patients will widened our knowledge of type A HE and help to find the clue of new therapy.

The present study was aimed to determine whether there is any abnormality in thyroid hormones among patients with type A HE compared to patients with acute liver injury (ALI) without encephalopathy.

## RESULTS

### Clinical characteristics of study population

**Figure 1:**
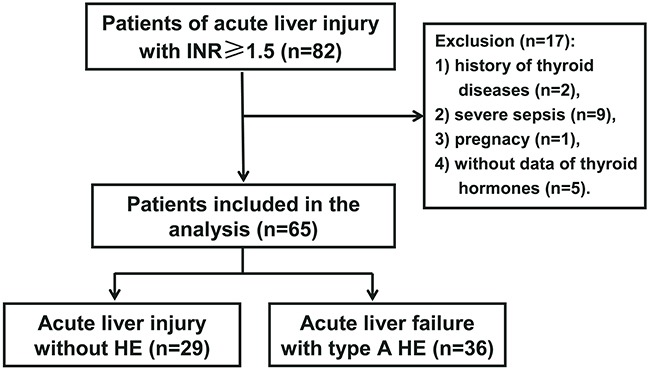
Flow chart for patients enrollment and evaluation HE: hepatic encephalopathy. There were 82 patients of ALI with INR≥1.5 hospitalized during the study period, and among them 65 patients were involved in the study, including 36 ALF patients with type A HE and 29 patients without HE

A total of 65 patients were involved in the analysis, including 36 ALF patients with type A HE and 29 ALI patients without encephalopathy as control (Figure 1). As depicted in Table 1, there was no difference in gender, age, etiology, alanine aminotransferase (ALT), albumin, and creatinine between patients with and without type A HE. However, patients with type A HE shown higher levels of aspartate aminotransferase (AST) (999 *vs* 487 U/L, *P* = 0.016), bilirubin (315 *vs* 249 μmol/L, *P* = 0.026), INR (3.13 *vs* 2.04, *P* < 0.001), and the MELD score (31 *vs* 26, *P* < 0.001) compared to patients without encephalopathy.

**Table 1:** Clinical characteristics of the study population

	Patients of acute liver injury with INR≥1.5		*P* value
	Without HE (n=29)	type A HE (n=36)	
Male^c^	22 (76%)	32 (89%)	0.196
Age (years)^a^	47±13	45±12	0.540
Etiology (viral)^c^	17 (77%)	22 (61%)	0.230
ALT (U/L)^b^	964 (1000)	1117 (1173)	0.874
AST (U/L)^b^	487 (641)	999 (1302)	0.016
Albumin (g/L)^a^	31.3±3.9	30.6±5.5	0.756
Bilirubin (μmol/L)^b^	249 (174)	315 (158)	0.026
INR^b^	2.04 (0.83)	3.13 (1.96)	<0.001
Creatinine (μmol/L)^b^	62 (25)	68(31)	0.373
MELD^a^	26±4	31±6	<0.001

^a^Variables are expressed as mean ± standard deviation, ^b^median (IQR), or ^c^n (%).

### Alteration of thyroid hormones in patients with type A HE

**Figure 2:**
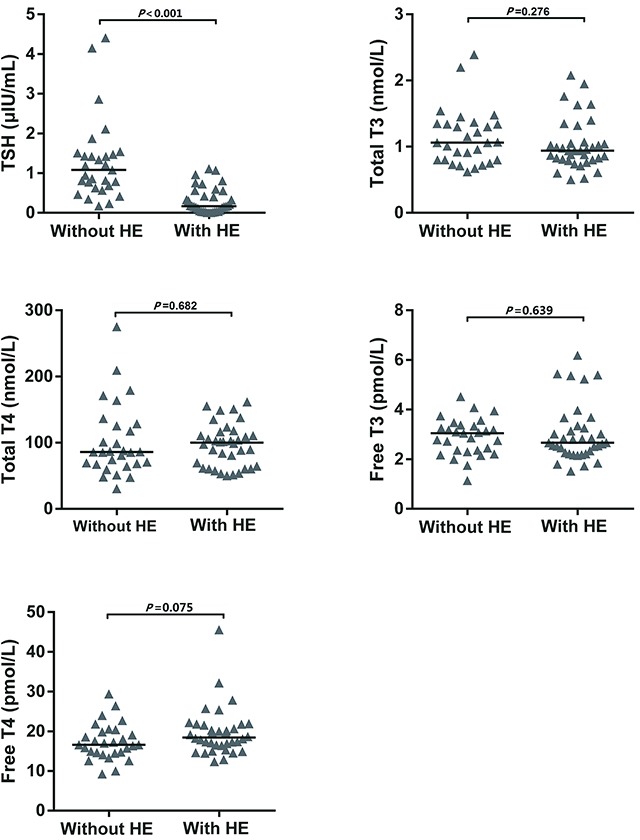
Levels of thyroid hormones between ALI patients without HE and ALF patients with type A HE Bars depicted median. There was significant difference in TSH level between patients with and without HE (*P*<0.001) **(A)**. There was no difference in the levels of total T4, total T3, free T4, and free T3 **(B–E)**.

Patients with type A HE showed subclinical central hypothyroidism: a condition of reduced TSH level with normal T3 and T4 levels. Compared to ALI patients without HE, patients with type A HE depicted significantly low TSH levels (1.08 *vs* 0.17 μIU/mL, *P*<0.001) (Figure 2A). There was no difference in the levels of total T4 (86 *vs* 100 nmol/L, *P*=0.682), total T3 (1.1 *vs* 0.9 nmol/L, *P*=0.276), free T4 (16.6 vs 18.5 pmol/L, *P*=0.075), and free T3 (3.1 vs 2.7 pmol/L, *P*=0.639) between the two groups (Figure 2B–2E and Table 2).

**Table 2:** Levels of thyroid hormones in patients with and without HE

Thyroid hormones	Patients of acute liver injury with INR ≥ 1.5		*P* value
	Without HE (n=29)	type A HE (n=36)	
TSH (μIU/mL)	1.08 (0.84)	0.17 (0.49)	<0.001
Total T4 (nmol/L)	86 (59)	100 (53)	0.682
Total T3 (nmol/L)	1.1 (0.5)	0.9 (0.2)	0.276
Free T4 (pmol/L)	16.6 (5.6)	18.5 (5.3)	0.075
Free T3 (pmol/L)	3.1 (1.0)	2.7 (1.1)	0.639

Variables are expressed as Median (IQR).

### Factors associated with occurrence of low TSH

In univariate logistic regression analysis, the occurrence of low TSH (<0.4 μIU/mL) was associated with HE, bilirubin level, INR, and MELD score. In multivariate analysis, HE (OR=12.32: 95% CI 2.85—53.18, *P*=0.001) still had significance, but bilirubin level, INR, and MELD score were statistically insignificant (Table 3).

**Table 3:** Evaluation of risk factors associated with low TSH level (< 0.4μIU/mL) by logistic regression analysis

Variables	univariate		*P* value	multivariate		*P* value
	OR	95% CI		OR	95% CI	
Gender (female)	0.44	0.10—1.82	0.254			
Age	0.99	0.95—1.03	0.46			
HE	39.20	4.79—320.70	0.001	12.32	2.85—53.18	0.001
ALT	1.000	1.000—1.001	0.325			
AST	1.000	0.999—1.000	0.269			
Albumin	1.09	0.928—1.281	0.294			
Bilirubin	1.006	1.023—1.010	0.023	1.003	0.994—1.012	0.541
INR	1.77	1.17—2.68	0.007	1.23	0.39—3.90	0.723
MELD	1.20	1.08—1.34	0.001	1.03	0.72—1.48	0.868
Creatinine	1.01	0.99—1.02	0.526			

OR, odds ratio; CI, confidence interval. Multivariate analysis identified that HE was the independent risk factor associated with the occurrence of low TSH level.

**Figure 3:**
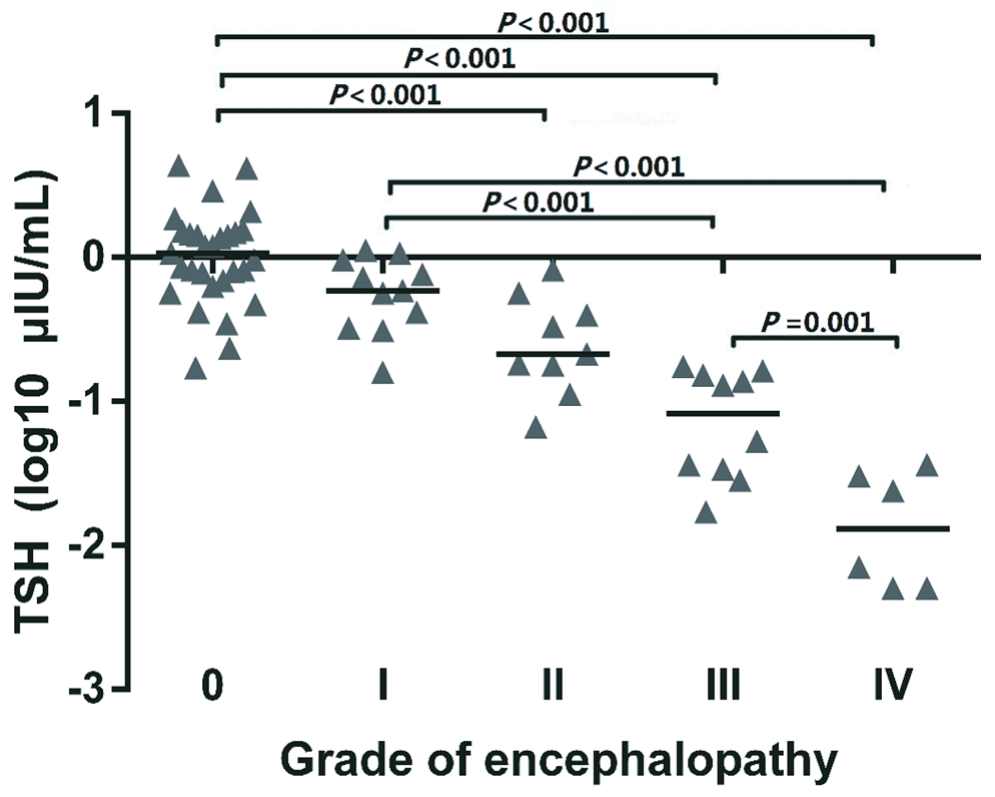
TSH levels in different grades of encephalopathy The data of TSH level were log-transformed. Patients with grade III—IV encephalopathy had significantly low levels of TSH compared to patients with 0—II grade.

TSH level was reduced with increase of encephalopathy grade, and the level was significantly lower in patients with grade III—IV encephalopathy than in patients with 0—II grade (Figure 3). The medians of TSH levels from grade 0 to IV were 1.08, 0.59, 0.21, 0.09, and 0.02 μIU/mL, respectively. Bivariate correlation analysis revealed an inverse correlation between TSH level and the grade of encephalopathy. The correlation remained statistically significant by partial correlation analysis (r =−0.795, *P* < 0.001) after adjusting for bilirubin, INR, MELD (Table 4).

**Table 4:** Evaluation of correlation between TSH level and clinical variables

TSH	Bivariate correlation		Partial correlation	
	r	*P*	r	*P*
Gender	0.150	0.234		
Age	0.148	0.240		
Grade of HE	−0.824	<0.001	−0.795	<0.001
ALT	−0.190	0.130		
AST	0.087	0.490		
Albumin	−0.113	0.453		
Bilirubin	−0.284	0.022	0.089	0.491
INR	−0.547	<0.001	0.153	0.235
MELD	−0.565	<0.001	−0.045	0.728

Partial correlation analysis showed that there was an inverse correlation between TSH level and the grade of encephalopathy.

### Increased mortality in patients with low TSH

**Figure 4:**
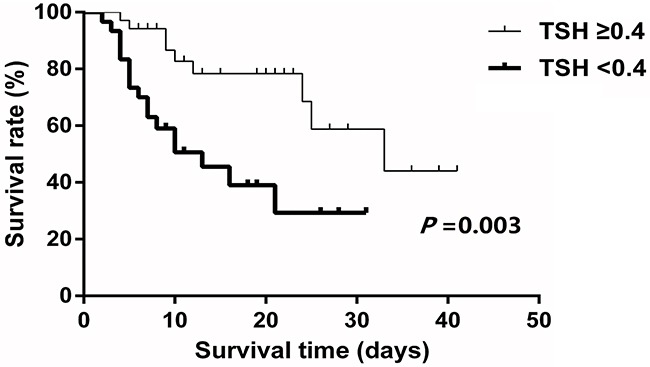
Survival rate between patients with and without low TSH Patients with low TSH had worse transplant-free survival compared to patients with normal TSH level (*P*=0.003).

A total of 21 patients died and 5 patients received liver transplantation during hospital stay. Patients with low TSH level had worse transplant-free survival rate compared to patients with normal TSH level (Figure 4). The transplant-free survival rate between patients with and without low TSH level were 29.3% *vs* 44.1% (*P* = 0.003) and the median survival time between two groups were 13 *vs* 33 days.

## DISCUSSION

The abnormality of thyroid hormones in patients with liver diseases was often reported. The “sick euthyroid” usually manifested as primary hypothyroidism, and the abnormalities were often presented as decreased levels of T3 and T4, and the TSH level is usually normal or slightly increased [9]. The “sick euthyroid” was associated with the severity of liver diseases and mortality of patients [4]. However, studies about the alteration of thyroid hormones in patients with HE were few.

There were some studies on thyroid hormones changes in patients with type C HE. They demonstrated that cirrhosis patients with type C HE had significantly lower levels of T3 and T4 compared to patients without encephalopathy, but there was no difference in TSH level. Given that liver impairment in type C HE patients was worse than patients without encephalopathy in these studies, the alteration of thyroid hormones in patients with type C HE might be related to the underlying deteriorated liver functions and the effect of type C HE on thyroid hormones was slight [5, 6].

This is the first study to determine the alteration of thyroid hormones in patients with type A HE. It was observed that the patients with type A HE had significantly reduced TSH level compared to ALI patients without HE, and there was no difference in the levels of T3 and T4 between the two groups. This subclinical central hypothyroidism in type A HE patients was different from the primary hypothyroidism reported among patients with type C HE. Furthermore, the TSH level depicted a negative correlation with the grade of encephalopathy in type A HE patients.

The underlying mechanism of the different alteration of thyroid hormones between type A and type C HE might be related to difference in pathogenesis. Type A HE is characterized by astrocyte swelling often leading to cerebral edema and its complications (intracranial hypertension, brain herniation) [9, 10]. The occurrence of cerebral edema and intracranial hypertension is related to severity of encephalopathy. Cerebral edema is seldom observed in patients with grade I-II encephalopathy, but increases to 25% to 35% with progression to grade III, and 65% to 75% or more in patients with grade IV encephalopathy [11]. However, type C HE is characterized by so-called Alzheimer type II change in the astrocytes and the occurrence of cerebral edema is seldom [12]. In the present study, logistic regression analysis revealed that type A HE was an independent related factor for the occurrence of low TSH. Correlation analysis also showed that there was a negative correlation between TSH level and the grade of encephalopathy. Therefore, the decrease of TSH level might be related with the impact of cerebral edema on functions of hypothalamus and pituitary caused by type A HE that resulted in subclinical central hypothyroidism.

It was also noticed that patients with low TSH had a significantly increased mortality rate compared to patients with normal TSH level. This could be due to the severity of encephalopathy and liver impairment in patients with reduced TSH level. It is not known whether restoration of the reduced thyroid levels could contribute to the recovery of patients. Hypothyroidism could minimize acute liver injury and chronic liver fibrosis due to hypometabolism [13–15]. However, it may affect for hepatocyte regeneration which is important for the recovery of patients with ALF [16–17]. It remains elusive whether TSH level will return to normal when type A HE resolved. No matter the decreased TSH levels in patients with type A HE is an adaptive response or a causative risk, it is a useful non-invasive marker for the severity of the disease and a poor prognosis indicator.

This is the first study to explore the abnormality of thyroid hormones in type A HE patients, and there are some limitations. The major weakness of our study is a retrospective observational research. Although we investigate the factors associated with the abnormality of thyroid hormones, literature survey and mechanistical investigation are not adequate. The present conclusions obtained by the retrospective method need to be further evaluated by prospective studies.

In conclusion, the results of the present study demonstrated that ALF patients with type A HE have significantly reduced TSH. A negative correlation was also observed between the reduced TSH level and the grade of HE. Furthermore, the decreased TSH was associated with mortality of patients.

## MATERIALS AND METHODS

### Patient population

In the retrospective study, we collected data of patients with severe acute liver injury hospitalized in the ICU department of Tianjin second people’s Hospital from October 2011 to December 2014. Inclusion criteria were: 1) ALI patients with an illness of < 26 weeks’ duration; 2) international normalized ratio (INR) ≥1.5; 3) age 18 years or older. Exclusion criteria were: 1) evidences of underlying systemic diseases; 2) history of thyroid diseases; 3) intake of drugs known to affect thyroid function; 4) severe sepsis; 5) absence of thyroid hormones testing; 6) pregnancy. A total of 65 patients were included. The etiology of patients included was; viral hepatitis, 48 patients; drug-induced, 11 patients; autoimmune hepatitis, 1 patient; and unknown etiology, 5 patients.

The study was approved by the Committee of Ethics in Tianjin second people’s Hospital and followed the principles expressed in the Declaration of Helsinki (revised 2013).

### Data collection

The clinical data of patients on admission were collected including demographic information, etiological examination, liver function tests, and thyroid hormone levels. Model for end-stage liver disease (MELD) score was calculated as: MELD = 3.8 × Ln (TBIL [mg/dl]) + 11.2 × Ln(INR) + 9.6 × Ln(Cr[mg/dl]) + 6.4 × etiology.

The thyroid hormones were measured by electro-chemiluminescence methods using standard auto-analyzer (Cobas 6000, Roche Diagnostics Corp, IN), including thyroid stimulating hormone (TSH), total triiodothyronine (total T3), total thyroxine (total T4), free triiodothyronine (free T3), and free thyroxine (free T4). Normal values of thyroid hormones in our laboratory are; TSH: 0.27–4.20 μIU/mL, total T4: 66–181 nmol/L, total T3: 1.3–3.1 nmol/L, free T4: 12–22 pmol/L, and free T3: 3.1–6.8 pmol/L. The low TSH is defined as a level less than 0.4 μIU/mL, according to U.S. Preventive Services Task Force recommendation statement [18].

### The diagnosis of ALF and type A HE

ALF was defined as acute liver function impairment with INR ≥1.5 and any degree of encephalopathy in a patient without pre-existing cirrhosis and with an illness of <26 weeks’ duration [11]. The diagnosis of HE was consistent with the definition established by the Working Party for Hepatic Encephalopathy in 1998 [7]. Encephalopathy was graded based on the West-Haven Criteria as follows: grade I characterized by confusion and mood changes, grade II-patient was drowsy or shows inappropriate behavior or has presence of asterixis, grade III-patient was sleepy but arousable, grade IV-patient was unresponsive to deep pain [19, 20].

All the data regarding treatment and outcome of patients during hospital stay were collected. Patients were provided with general management, such as metabolic support, maintaining adequate hemodynamics, correction of coagulopathy, and surveillance for infection. Etiology management included nucleos(t)ide analogues for viral hepatitis B, corticosteroid treatment for suspected autoimmune hepatitis, and discontinue of medications that produce drug-induced hepatotoxicity. In patients with HE, oral lactulose and intravenous L-ornithine L-aspartate were given for treatment of hyperammonemia, and mannitol and glycerin fructose were used if cerebral edema and intracranial hypertension occurred. Patients received prognostic evaluation repeatedly to identify candidates for liver transplantation. Data of adverse events and time of death were collected.

### Statistical analysis

Normally distributed continuous variables were reported as Mean ± SD and were compared by Student’s *t* test. Non-normally distributed continuous data were reported as median with interquartile ranges (IQR) and were compared by Mann-Whitney U test. Categorical variables were reported as percentage and were compared by Chi-Square test or Fisher’s exact test. Logistic regression analysis was employed to identify factors associated with low TSH level. Correlation coefficient was calculated using Spearman’s rank order, and partial correlation analysis was performed to evaluate the adjusted association between TSH level and the grade of encephalopathy. Survival analysis was conducted using the Kaplan–Meier method, and the log-rank test was used to compare survival curves. A two-tailed *P* value < 0.05 was considered as statistically significant. SPSS 20.0 software (SPSS, Inc., Chicago, IL) was used for all statistical analyses.

## Abbreviations

HE: hepatic encephalopathy
ALF: acute liver failure
ALI: acute liver injury
TSH: thyroid stimulating hormone
T3: triiodothyronine
T4: thyroxine
ALT: alanine aminotransferase
AST: aspartate aminotransferase
INR: international normalized ratio
MELD: model for end-stage liver disease
OR: odds ratio
CI: confidence interval
